# Extensive preclinical evaluation of lutetium-177-labeled PSMA-specific tracers for prostate cancer radionuclide therapy

**DOI:** 10.1007/s00259-020-05057-6

**Published:** 2020-10-23

**Authors:** Eline A. M. Ruigrok, Nicole van Vliet, Simone U. Dalm, Erik de Blois, Dik C. van Gent, Joost Haeck, Corrina de Ridder, Debra Stuurman, Mark W. Konijnenberg, Wytske M. van Weerden, Marion de Jong, Julie Nonnekens

**Affiliations:** 1 Department of Radiology and Nuclear Medicine, Erasmus MC, Rotterdam, The Netherlands; 2 Department of Experimental Urology, Erasmus MC, Rotterdam, The Netherlands; 3 Department of Molecular Genetics, Erasmus MC, Rotterdam, The Netherlands; 4 Oncode Institute, Erasmus MC, Rotterdam, The Netherlands; 5 Erasmus MC, Room Ee757R, PO box 2040, 3000 CA Rotterdam, The Netherlands

**Keywords:** PSMA, Prostate cancer. Targeted radionuclide therapy, Preclinical research

## Abstract

**Purpose:**

Various radiolabeled prostate-specific membrane antigen (PSMA)–targeting tracers are clinically applied for prostate cancer (PCa) imaging and targeted radionuclide therapy. The PSMA binding affinities, biodistribution, and DNA-damaging capacities of these radiotracers have not yet been compared in detail. A major concern of PSMA-targeting radiotracers is the toxicity in other PSMA-expressing organs, such as the salivary glands, thus demanding careful evaluation of the most optimal and safest radiotracer. In this extensive preclinical study, we evaluated the clinically applied PSMA-targeting small molecule inhibitors DOTA-PSMA-617 (PSMA-617) and DOTAGA-PSMA-I&T (PSMA-I&T) and the PSMA nanobody DOTA-JVZ-007 (JVZ-007) using PSMA-expressing cell lines, a unique set of PCa patient-derived xenografts (PDX) and healthy human tissues.

**Methods and results:**

In vitro displacement studies on PSMA-expressing cells and cryosections of a PSMA-positive PDX revealed high and specific binding affinity for all three tracers labeled with lutetium-177 with IC_50_ values in the nanomolar range. Interestingly, [^177^Lu]Lu-JVZ-007 could not be displaced by PSMA-617 or PSMA-I&T, suggesting that this tracer targets an alternative binding site. Autoradiography assays on cryosections of human salivary and renal tissues revealed [^177^Lu]Lu-PSMA-617 to have the lowest binding to these healthy organs compared with [^177^Lu]Lu-PSMA-I&T. In vivo biodistribution assays confirmed the in vitro results with comparable tumor uptake of [^177^Lu]Lu-PSMA-617 and [^177^Lu]Lu-PSMA-I&T at all timepoints, resulting in induction of similar levels of DNA double-strand breaks in the tumors. However, [^177^Lu]Lu-PSMA-I&T demonstrated approximately 40× higher renal uptake at 4 and 8 h post injection resulting in an unfavorable tumor-to-kidney ratio.

**Conclusion:**

[^177^Lu]Lu-PSMA-617 has the most favorable biodistribution in mice as well as more favorable binding characteristics in vitro in PSMA-positive cells and human kidney and salivary gland specimens compared with [^177^Lu]Lu-PSMA-I&T and [^177^Lu]Lu-JVZ-007. Based on our preclinical evaluation, [^177^Lu]Lu-PSMA-617 is the best performing tracer to be taken further into clinical evaluation for PSMA-targeted radiotherapeutic development although with careful evaluation of the tracer binding to PSMA-expressing organs.

**Electronic supplementary material:**

The online version of this article (10.1007/s00259-020-05057-6) contains supplementary material, which is available to authorized users.

## Introduction

Prostate cancer (PCa) is a major public health problem. In 2018, prostate cancer (PCa) was diagnosed approximately 450,000 times in Europe and had caused an estimated 107,000 deaths [1, 2]. Patients with metastasized PCa are currently treated with systemic therapy consisting of androgen deprivation therapy and chemotherapy or combinations thereof. Despite major improvements in therapy options, these patients show a 5-year survival rate of approximately 30%, underscoring the need for alternative approaches [3].

Prostate-specific membrane antigen (PSMA) is a type II transmembrane enzymatic protein which is overexpressed in 90–100% of PCa cases. High PSMA expression levels are found in hormone-resistant tumors and metastases indicating that PSMA expression is correlated to disease progression [4–6]. This makes PSMA an ideal target for theranostics. PSMA-positron emitting tomography scans have shown PCa (oligo)metastases that were untraceable with conventional methods which logically led to the development of PSMA-specific tracers suitable for targeted radionuclide therapy (TRT). The excellent theranostic application of PSMA-targeting compounds resulted in the development of various tracers of which the most promising were tested in clinical trials; these are showing promising results [7–9]. However, the expression of PSMA in healthy organs, such as the small intestine, central nervous system, proximal renal tubules, prostate, and especially the salivary and lacrimal glands, can lead to serious side effects, which may strongly affect the quality of life of the treated patients [10–12]. Protection of the kidneys and salivary and lacrimal glands is the principal challenge for PSMA-TRT, because these organs show high uptake of almost all PSMA-targeting radiotracers. Predominantly salivary gland toxicity has been reported as dose-limiting, causing a significant decrease in the quality of life of PSMA-TRT-treated patients [13].

The majority of PSMA-targeting tracers that are being used for clinical and preclinical research are small molecule inhibitors. To date, DOTA-PSMA-617 and DOTAGA-PSMA-I&T (further referred to as PSMA-617, PSMA-I&T) are the most often applied small molecule inhibitors for PSMA-TRT and PCa diagnostics. These tracers share the same PSMA-binding motif, and both comprise a DOTA or DOTAGA chelator that can be labeled with therapeutic or diagnostic radionuclides, respectively [9, 14, 15]. Currently, lutetium-177-labeled PSMA-I&T and PSMA-617 are being examined separately in a phase II and phase III clinical trial [16, 17]. Because both tracers have never extensively been compared in the same study, neither clinically nor preclinically, it remains to be elucidated which PSMA-targeting tracer is the best in terms of tumor-targeting abilities and induction of side effects. A proper evaluation is therefore essential, however currently missing. To our knowledge, this study is the first preclinical evaluation and comparison of the binding affinities, biodistribution, and DNA-damaging capacity of the clinically applied PSMA-targeting small molecule inhibitors PSMA-617 and PSMA-I&T. Additionally, PSMA nanobody JVZ-007 was included in this comparison as it previously showed promising tumor-specific binding properties [18].

## Material and methods

### Cell culture

Experiments were performed on LNCaP, DU145-PSMA (two subclones #15 and #18), and U2OS-PSMA cells. DU145 and U2OS cells were transfected with a PSMA plasmid kindly provided by Dr. Giulio Fracasso, University of Verona, to create the stably PSMA-expressing cell lines. LNCaP and DU145-PSMA were cultured in RPMI 1640, Glutamax medium (Gibco) supplemented with 10% fetal bovine serum (Gibco), penicillin (100 units/mL) (Gibco), and streptomycin (100 μg/mL) (Gibco). U2OS-PSMA cells were cultured with DMEM, Glutamax medium supplemented with 10% fetal bovine serum, penicillin (100 units/mL), and streptomycin (100 μg/mL). All cells were grown at 37 °C and 5% CO_2_.

### Patient-derived xenograft tumors of human PCa

A patient-derived xenograft (PDX) panel of human PCa has been established from fresh human PCa samples by subcutaneous transplantation in NMRI nu/nu mice. The PDX is serially passaged as previously described [19]. This well-characterized PDX panel includes PSMA-positive PC295, PC310, and PC82, and PSMA-negative PC324. All conducted animal experiments were approved by the Erasmus MC Animal Welfare Committee and were in accordance to the European law. NMRI (Foxn1 nu/nu) male animals (Janvier) were maintained in standard 12-h light/dark cycle with water and food ad libitum.

### Radiolabeling

PSMA-617 (Advanced Biochemical Compounds) and JVZ-007 both contain a 1,4,7,10-tetra-azacycloododecane-N,N′,N″,N‴-tetraacetic acid (DOTA) chelator. PSMA-I&T (kindly provided by Prof. Dr. Hans-Jürgen Wester, Technische Universität München) contains a 1,4,7,10-tetraazacyclododececane,1-(glutaric acid)-4,7,10-triacetic acid (DOTAGA) chelator. All tracers were labeled with lutetium-177 (LuMark, IDB Holland). Labeling of PSMA-617, PSMA-I&T, and JVZ-007 was performed as previously described [18, 20–22]. Quenchers (3.5 mM ascorbic acid, 3.5 mM gentisic acid, 10 mM methionine) (Sigma-Aldrich) were added to prevent radiolysis of the radiotracer. For all in vitro assays, the tracers were labeled with a molar activity of 40 MBq/nmol. The in vivo biodistribution assay was performed with [^177^Lu]Lu-PSMA-617 and [^177^Lu]Lu-PSMA-I&T with a molar activity of 100 MBq/nmol. Quality control was assessed using high-pressure liquid chromatography and instant thin-layer chromatography (ITLC-SG). For all labelings, the radiochemical yield was > 95% and the radiochemical purity was > 90%.

### In vitro autoradiography assays

Fresh frozen tissues (human salivary gland, human kidney, murine kidney, and PDX PC295) were sectioned into 10-μm-thick slices and mounted on starfrost glass slides (Thermo Scientific). To prevent nonspecific binding, the slides were incubated for 10 min at room temperature (RT) with washing buffer (167 mM Tris-HCl, 5 mM MgCl_2_) containing 0.25% bovine serum albumin (BSA) whereafter the slides were incubated with one of the three [^177^Lu]Lu-labeled tracers for 1 h at RT. The slides were drained off and washed. After air drying, super resolution phosphor screens (Perkin Elmer) were exposed to the slides for 24 h after which the screens were read using the Cyclone (Perkin Elmer), and analyzed and quantified using Optiquant software.

For the IC_50_ binding affinity assays, tissue slices of PDX PC295 were incubated with 100 μL of each 10^−9^ M [^177^Lu]Lu-labeled tracer with increasing concentrations (10^−13^ M–10^−6^ M) of unlabeled compound in triplicate. All experiments were performed twice.

For binding assays, PSMA-positive PDXs PC295, PC310, and PC82 and PSMA-negative PDX PC324 [23] and 16 human kidney and 6 salivary gland samples from healthy individuals (obtained from the Erasmus MC tissue bank) were incubated with 100 μL of each 10^−9^ M [^177^Lu]Lu-labeled tracer with and without 10^−6^ M unlabeled compounds to determine specificity. The binding assay was performed in triplicate.

### In vitro uptake assays

IC_50_ displacement assays were performed on LNCaP, DU145-PSMA, and U2OS-PSMA cells which were incubated with the three [^177^Lu]Lu-labeled tracers (10^−9^ M) together with increasing concentrations (10^−13^ M–10^−6^ M) of unlabeled compounds in triplicate. All experiments were performed twice.

One day prior to the experiment, 50,000 cells were seeded in 12-well plates. Adherent cells were incubated with the radiolabeled tracers for 3 h at 4 °C to prevent internalization. After incubation, the incubation medium containing the radiotracer was removed, and cells were washed twice with phosphate-buffered saline (PBS) (Gibco) whereafter the cells were lysed using 0.1 M NaOH. The cell lysate was collected and measured using a γ-counter (1480 WIZARD automatic γ counter; PerkinElmer). The data are expressed as percentage of the added activity per 100,000 cells (%AA/100,000 cells). Cells were counted using an automated CASY cell counter (Bioké).

### In vivo biodistribution studies and ex vivo autoradiography assay

Mice were subcutaneously transplanted with a small fragment of PC295 PDX on the right shoulder. A testosterone pellet (Applichem) was implanted subcutaneously on the right flank for optimal tumor take. Tumors were grown for 4–5 weeks to an average volume of 310 ± 111 mm^3^. When tumors reached the desired volume, animals were intravenously (i.v.) injected with 200 μL of 30 MBq/300 pmol [^177^Lu]Lu-labeled PSMA-617 or PSMA-I&T diluted in phosphate-buffered saline, with (*n* = 2 per group) or without (*n* = 4 per group) 100× excess of the same unlabeled compound. At 4, 8, and 24 h post injection (p.i.), animals were sacrificed and blood, tumor, pancreas, liver, spleen, small intestine, colon, adrenals, kidneys, prostate (anterior), lungs, salivary glands, muscle, bone (femur), and tail were collected. The tumors and one of the kidneys were cut in half and one-half was snap frozen in liquid nitrogen for autoradiography studies. The other half and all other organs were weighed, placed in formalin, and counted in a γ-counter. The amount of radioactivity that was taken up by the tumor and organs was determined and expressed as percentage injected activity per gram of tissue (%IA/g). Tumors and organs from animals that were not injected with the radiotracers were used as controls further in autoradiography and immunofluorescent staining studies.

For visualization of tracer biodistribution, single-photon emission computed tomography and computed tomography (SPECT/CT) scans were acquired in an additional group of mice (*n* = 3 per tracer). Each animal was scanned three times, at 4, 8, and 24 h p.i., under isoflurane/O_2_ anesthesia. Images were acquired using the VECTor/CT (MiLABs) in 40 min using the XXUHS collimator to image the whole body (transaxial fov 8 cm) in listmode using 94 bed positions. Images were reconstructed at 0.8 mm^3^ resolution with a 1 mm FWHM Gaussian post-filter using MiLABs REC8.0 software utilizing the SROSEM method with 9 iterations and maximally 128 subsets and triple energy settings for background correction (energy window settings 113 ± 11 kev, 210 ± 20 kev). After the final scan, animals were sacrificed, and organs were collected as described above. Data from these animals were added to the biodistribution data points of 24 h.

Snap frozen kidneys and tumor tissue were cryosectioned into 10 μm thick sections 24–48 h after organ retrieval. Sections were exposed to super resolution phosphor screens for 24 h. The level of bound radioactive tracer was analyzed and quantified as previously mentioned.

### Dosimetry

Data obtained during the in vivo distribution studies were used to calculate the absorbed dose per administered activity (30 MBq) for each organ according to the MIRD scheme [24]. The time-integrated activity coefficients were determined by fitting single-exponential curves to the activity concentration data points at 4, 8, and 24 h p.i. and integrating these curves over time. Least squares fitting was performed with the Graphpad Prism software. The S-values for lutetium-177 in a 28-g weight mouse were kindly provided by Dr. Erik Larsson. [25] The standard organ weights from this model were used to convert from activity concentration to activity. The absorbed dose in most organs could be calculated with this model, including the source contribution by the blood contents in bone marrow, for which a generic model was taken for the relative blood distribution in mice [26]. S-values for organs that were not included in the mouse model, such as adrenals, prostate, and salivary glands, were determined with the spherical node model within the Olinda/EXM code [27].

### Immunofluorescent staining and analysis

DNA damage was analyzed on the formalin-fixed paraffin-embedded (FFPE) 4 μm thick sections of the PC295 tumors of the in vivo biodistribution study. First, the sections were deparaffinized and rehydrated. Next, antigens were retrieved by heating the slides in Sodium Citrate buffer (pH 6.0). Sections were washed with PBS/Triton (0.025%) and blocked using 5% BSA in PBS/Tween (0.05%) + 0.3 M glycine for 60 min at RT. Subsequently, the sections were incubated overnight at 4 °C with the primary antibodies, anti-gH2AX (1:500, mouse, Millipore clone JBW301) and anti-53BP1 (1:1000, rabbit, Novus Biologicals NB100-904). Hereafter, the sections were washed using PBS/Tween (0.05%) and incubated with the secondary antibodies, goat anti-rabbit Alexa Fluor 488 and goat anti-mouse Alexa Fluor 594 (1:1000, Thermo Fisher Scientific) for 60 min at RT. Sections were then washed with PBS/Tween (0.05%) and mounted with Vectashield + Dapi (Vectorlabs). Fluorescent imaging was performed using a LSM700 confocal microscope (Zeiss). The images were analyzed using Image J software with automated DAPI segmentation and Find Maxima function. The average number of fluorescent foci per nucleus was determined for five random field images (40–50 nuclei per image) of each tumor.

### Statistics

Statistical analysis was performed using Graphpad Prism software (version 6.01). IC_50_ values were determined by plotting IC_50_ curves (non-linear regression) on the normalized data points. Significant differences were evaluated using either the 2-way ANOVA test followed by Sidak’s multiple comparisons tests or unpaired *t* test. A *P* value below 0.05 was considered significant.

## Results

### In vitro displacement assays revealed IC_50_ values in the nanomolar range for all three tracers

**Fig. 1:**
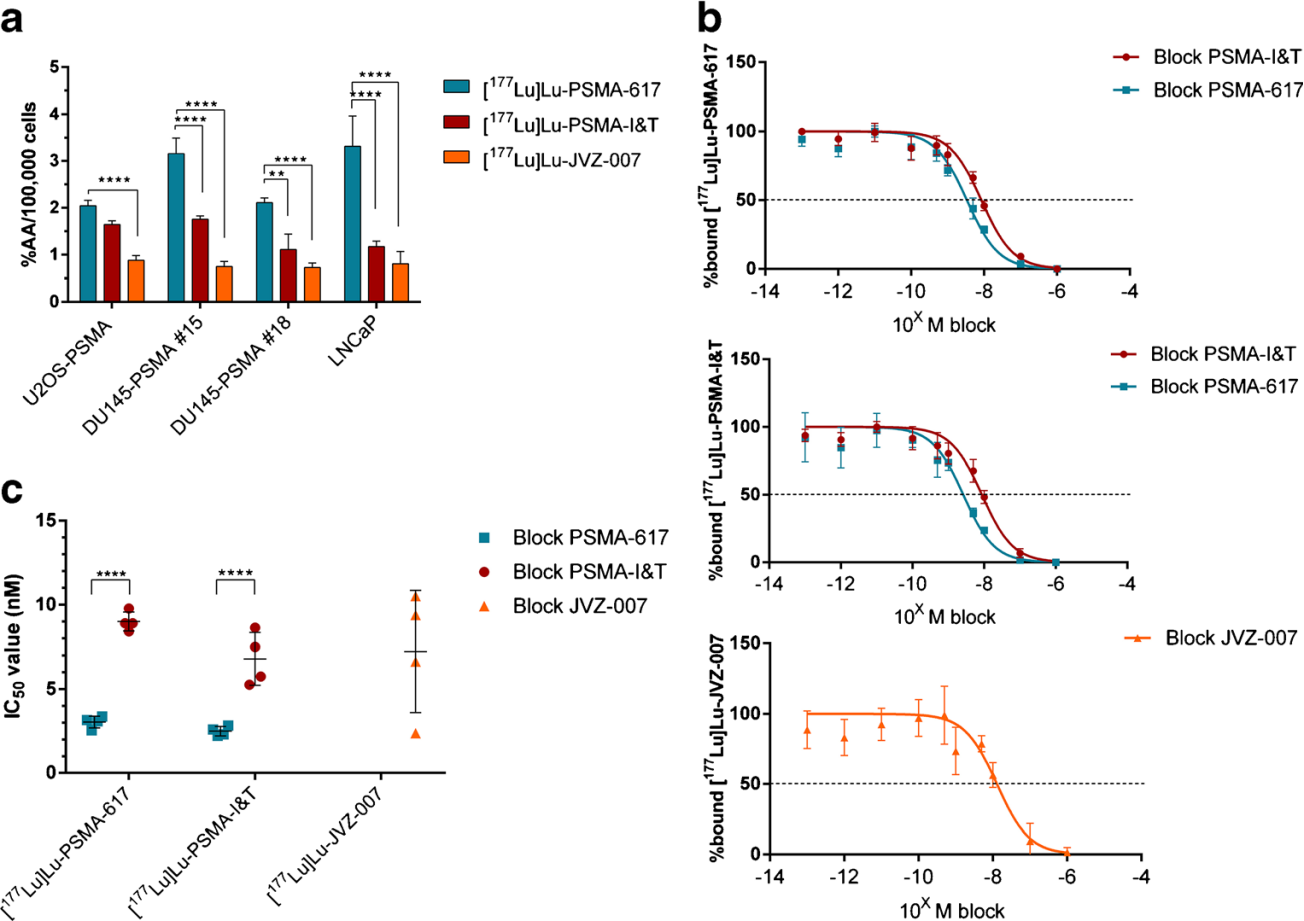
In vitro uptake determining binding and IC_50_ values of lutetium-177-labeled PSMA-617, PSMA-I&T, and nanobody JVZ-007. **a** Uptake (expressed in percentage of the added activity (%AA)) of the different tracers per 100,000 cells in four PSMA-expressing cell lines. **b** IC_50_ curves of in vitro displacement assays using DU145-PSMA #15 cells (IC_50_ curves of the other three cell lines can be found in the supplemental data (Suppl. Fig. 1)) **c** IC_50_ values of all in vitro uptake assays, each dot represents a different PSMA-expressing cell line. The error bars represent the standard deviation. Asterisks indicate significance (**P ≤ 0.01, ****P ≤ 0.0001)

In vitro uptake and autoradiography studies were performed to assess the binding of lutetium-177-labeled PSMA-617, PSMA-I&T, and JVZ-007. Uptake experiments using the PSMA transfected cell lines U2OS-PSMA and DU145-PSMA (both subclones #15 and #18) and the endogenous PSMA-expressing LNCaP revealed that [^177^Lu]Lu-PSMA-617 had the highest overall uptake, while the nanobody [^177^Lu]Lu-JVZ-007 showed the lowest uptake in all four cell lines (Fig. 1a). In vitro uptake displacement studies were performed in order to determine the IC_50_ values of the unlabeled compounds. Increasing concentrations of all three unlabeled tracers were used to block either [^177^Lu]Lu-PSMA-617, [^177^Lu]Lu-PSMA-I&T, or [^177^Lu]Lu-JVZ-007. PSMA-617 and PSMA-I&T could successfully block both [^177^Lu]Lu-PSMA-617 and [^177^Lu]Lu-PSMA-I&T with comparable IC_50_ values in the nanomolar range in all four PSMA-positive cell lines (Fig. 1 b, c). Increasing concentrations of JVZ-007 did not lead to displacement of lutetium-177-labeled PSMA-617 or PSMA-I&T. Similarly, increasing concentrations of any of the small molecule inhibitors did not lead to displacement of radiolabeled JVZ-007.

**Fig. 2:**
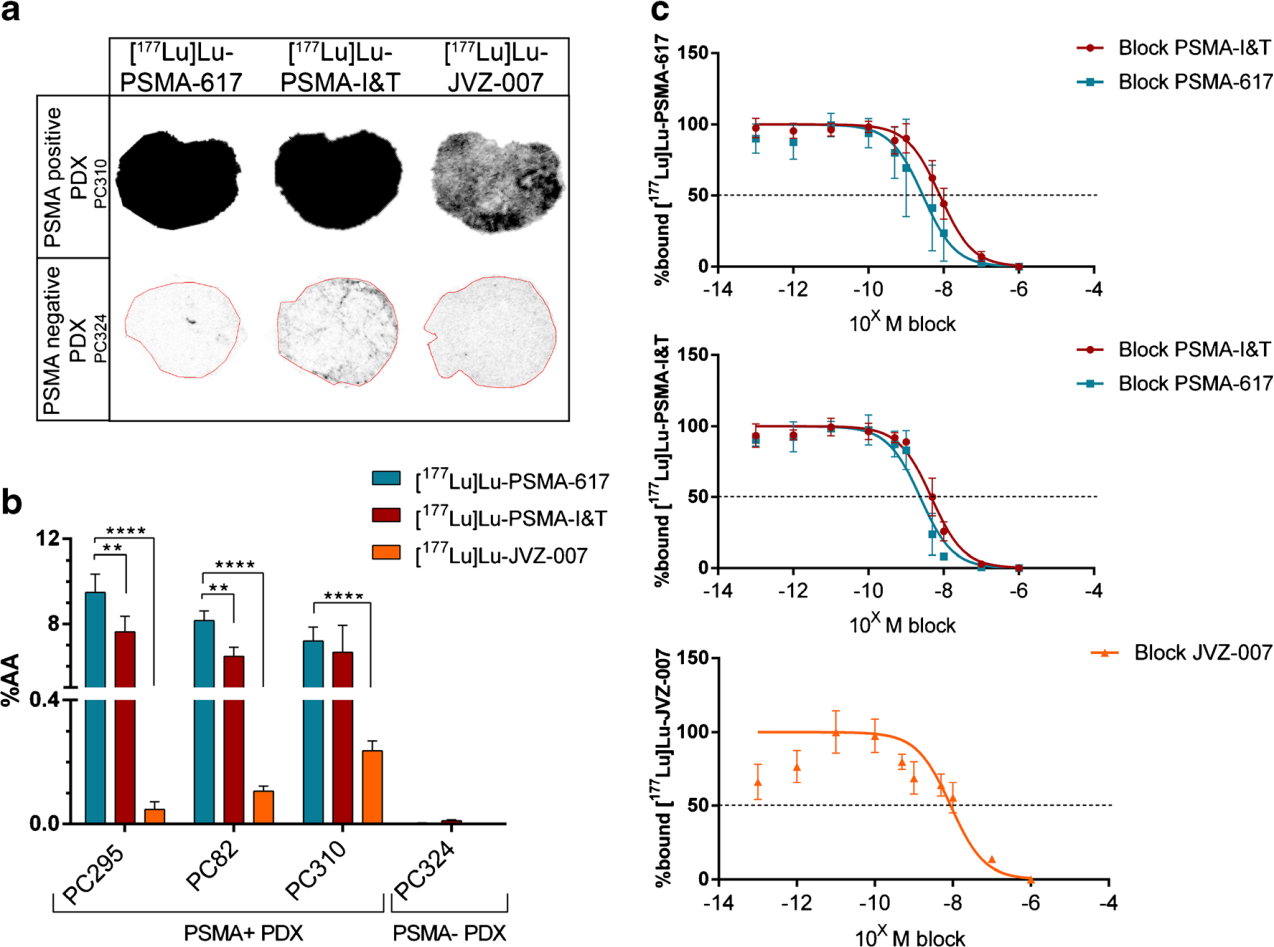
In vitro autoradiography assays determining binding and IC_50_ values of lutetium-177-labeled PSMA-617, PSMA-I&T, and nanobody JVZ-007. **a** Representative images of the autoradiography results on PC310 (PSMA-positive) and PC324 (PSMA-negative) PDX. **b** Quantification of the autoradiography results representing the total binding (percentage added activity (%AA)) of the radiotracers on PSMA-positive and PSMA-negative PDXs. **c** IC_50_ curves of autoradiography displacement assays using the PC295 PDX. The error bars represent the standard deviation. Asterisks indicate significance (**P ≤ 0.01, ****P ≤ 0.0001)

To validate the binding and IC_50_ values of the tracers in tumor tissues, we performed in vitro autoradiography studies on the PSMA-positive PDXs PC295, PC82, and PC310 (Fig. 2a, b). [^177^Lu]Lu-PSMA-617 and [^177^Lu]Lu-PSMA-I&T showed high binding to all three PSMA-positive PDXs, while nanobody [^177^Lu]Lu-JVZ-007 showing relatively low binding to these PDXs. No binding to the PSMA-negative PDX PC324 was observed for all three tracers, confirming their PSMA specificity. In vitro autoradiography displacement studies revealed IC_50_ values in the nanomolar range on the PSMA-positive PDX PC295, which were comparable with the IC_50_ values found in cells (Fig. 2c). Similar to the in vitro uptake study, addition of increasing concentrations of unlabeled PSMA-617 and PSMA-I&T to [^177^Lu]Lu-JVZ-007 did not lead to displacement of the radiolabeled nanobody and JVZ-007 was not able to block binding of [^177^Lu]Lu-PSMA-617 and [^177^Lu]Lu-PSMA-I&T.

### In vitro autoradiography studies revealed significantly higher binding of [^177^Lu]Lu-PSMA-I&T compared with [177Lu]Lu-PSMA-617 in human kidney and salivary gland tissue

**Fig. 3:**
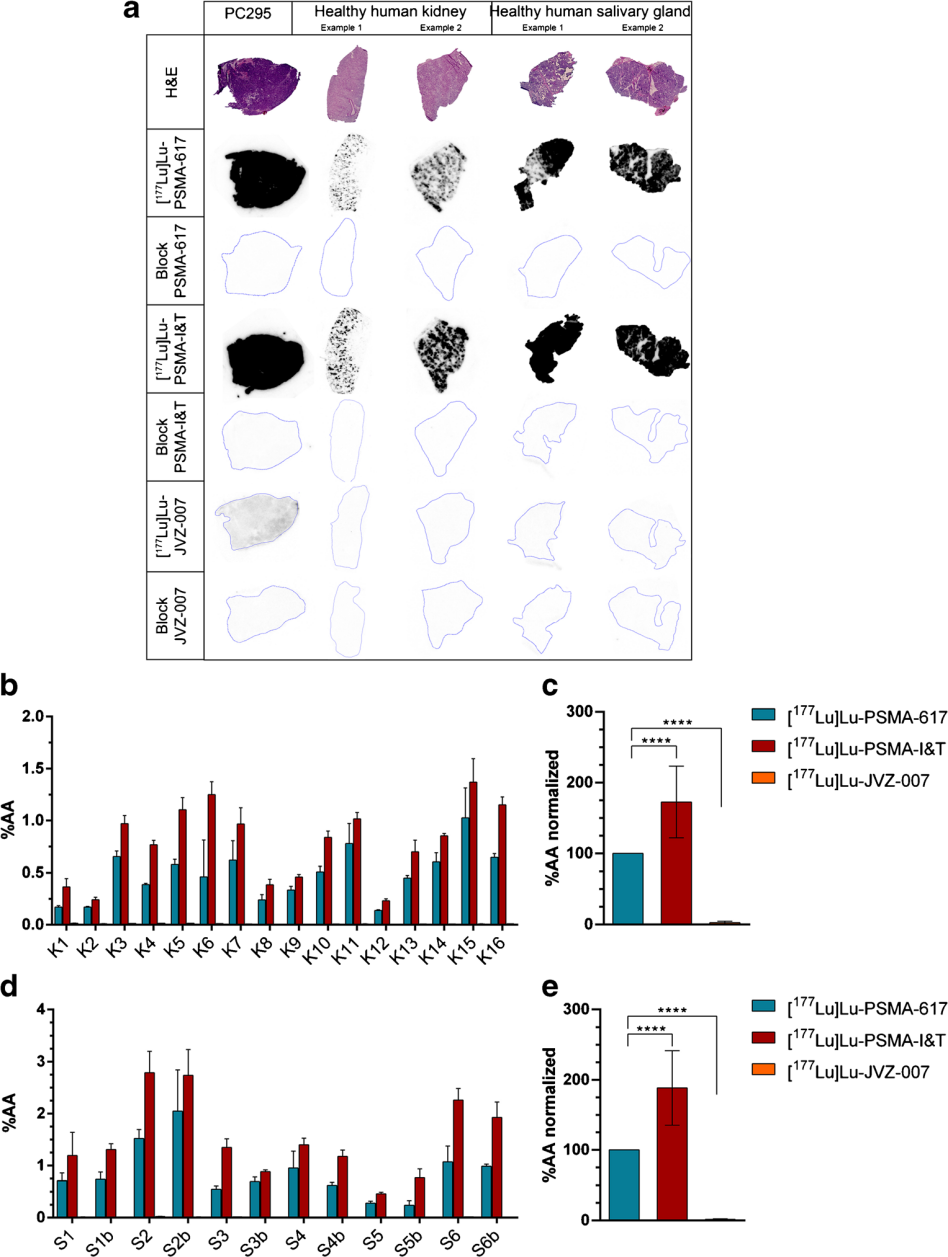
In vitro autoradiography assays determining the binding of lutetium-177-labeled PSMA-617, PSMA-I&T, and JVZ-007 to human kidney (K), salivary gland (S), and PC295 PDX cryosections. Samples of different patients (indicated with the different numbers) were used. **a** Representative images of the autoradiography results and corresponding H&E staining. **b** Autoradiography results quantified on human kidney tissue and **c**) when normalized for lutetium-177-labeled PSMA-617. **d** Autoradiography results quantified on human salivary gland tissue (‘b’ on the *x*-axis indicates separate analysis from the same patient) and **e** when normalized for lutetium-177-labeled PSMA-617. Asterisks indicate significance (****P ≤ 0.0001). All error bars indicate standard deviation

Autoradiography assays on healthy tissues were performed to compare the physiological binding of lutetium-177-labeled PSMA-617, PSMA-I&T, and JVZ-007. The autoradiography assay revealed relatively high level of [^177^Lu]Lu-PSMA-617 and [^177^Lu]Lu-PSMA-I&T binding to the healthy tissues, while the level of binding of [^177^Lu]Lu-JVZ-007 was much lower. An excess of unlabeled compound (block) inhibited binding of the radiotracers in all tested tissues indicating specificity of binding (Fig. 3a). [^177^Lu]Lu-JVZ-007 demonstrated a significantly lower level of binding to all tissues compared with both small molecule inhibitors (Fig. 3b, d). While [^177^Lu]Lu-PSMA-617 and [^177^Lu]Lu-PSMA-I&T both demonstrated similar high level of binding to the PC295 PDX positive control, [^177^Lu]Lu-PSMA-I&T presented with significantly higher level of binding to the human kidney and salivary gland tissue compared with [^177^Lu]Lu-PSMA-617 (Fig. 3c, e). Because of the low in vitro tumor binding of [^177^Lu]Lu-JVZ-007, the nanobody was excluded for further in vivo analysis.

### [^177^Lu]Lu-PSMA-I&T showed significantly higher kidney uptake and similar tumor uptake in vivo compared with [^177^Lu]Lu-PSMA-617

**Fig. 4:**
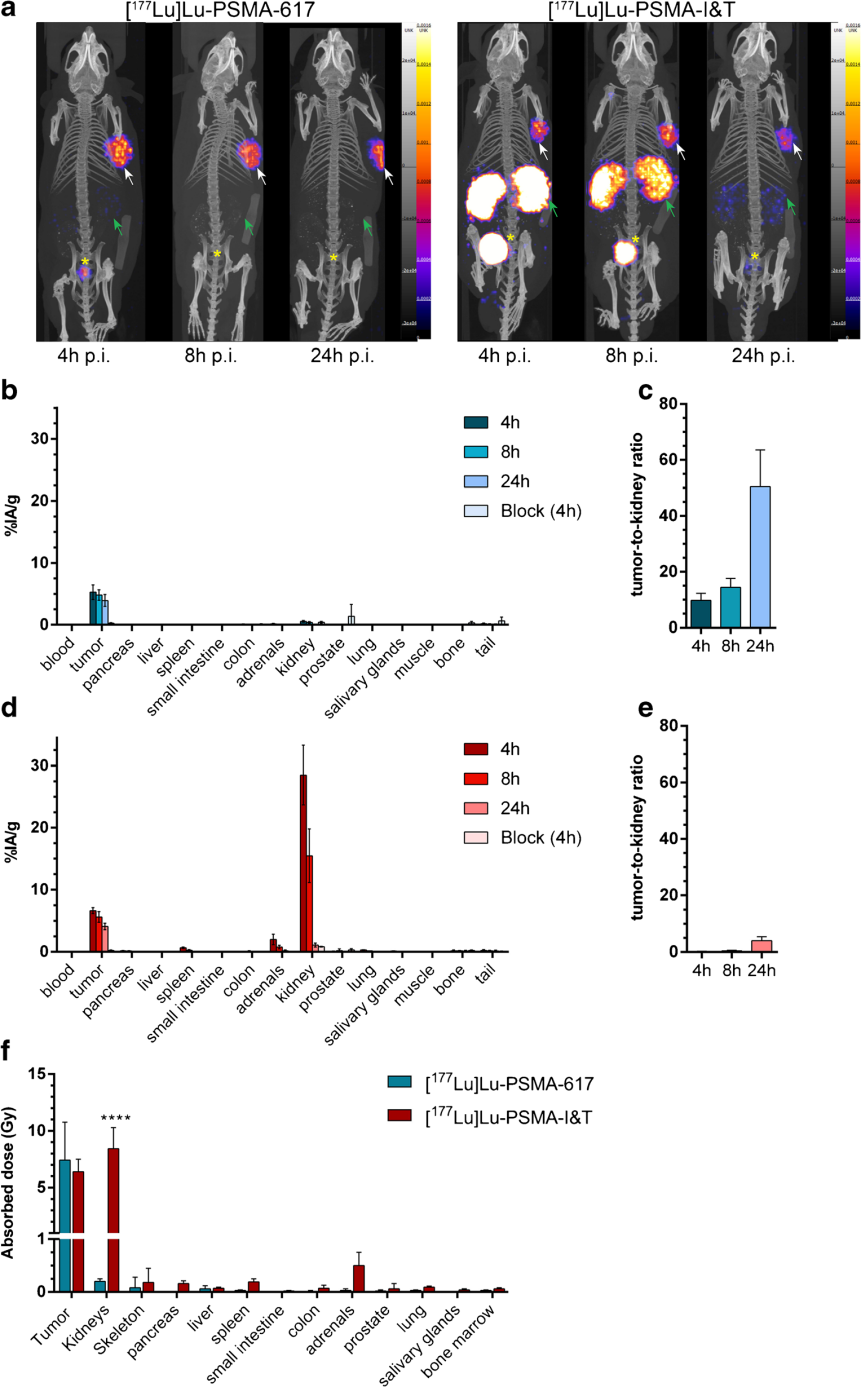
In vivo biodistribution of [^177^Lu]Lu-PSMA-617 and [^177^Lu]Lu-PSMA-I&T in mice bearing PC295 PDX. **a** Representative SPECT/CT images at 4, 8, and 24 h p.i. White arrows indicate the PC295 PDX, green arrows the kidneys, and the yellow asterisks indicate the bladder. **b** Biodistribution of [^177^Lu]Lu-PSMA-617 (expressed in percentage of injected activity per grams of tissue (%IA/g)) and **c** corresponding tumor-to-kidney ratio (error bars indicate standard deviation). **d** Biodistribution of [^177^Lu]Lu-PSMA-I&T and **e** corresponding tumor-to-kidney ratio (for **a**–**d**: error bars indicate standard deviation). **f** Absorbed dose per 30 MBq (error bars indicate standard error of the mean). Asterisks indicate significant differences between tracers (****P ≤ 0.0001)

In vivo experiments using PC295 PDX tumor-bearing mice were performed to compare the distribution of [^177^Lu]Lu-PSMA-617 and [^177^Lu]Lu-PSMA-I&T at three timepoints by SPECT and ex vivo biodistribution (Fig. 4a–e). [^177^Lu]Lu-PSMA-617 showed high tumor uptake at 4 h p.i. and at 8 and 24 h p.i. the uptake in the tumor slightly decreased. Renal uptake of [^177^Lu]Lu-PSMA-617 was the highest at 4 h p.i (Fig. 4b) with high tumor-to-kidney ratios at all measured timepoints (Fig. 4c). [^177^Lu]Lu-PSMA-I&T showed a similar although slightly higher tumor uptake compared with [^177^Lu]Lu-PSMA-617 at all timepoints. Renal uptake of [^177^Lu]Lu-PSMA-I&T was, however, significantly higher compared with that of [^177^Lu]Lu-PSMA-617 at 4 and 8 h p.i (Fig. 4d). Consequently, less favorable tumor-to-kidney ratios were reached with [^177^Lu]Lu-PSMA-I&T (Fig. 4e). No notable uptake was observed in any other organs for both radiolabeled tracers (Fig. 4a, b, d) except for the uptake of [^177^Lu]Lu-PSMA-I&T in the adrenal glands observed 4 h p.i.

The absorbed dose in tumor tissue was comparable for [^177^Lu]Lu-PSMA-617 and [^177^Lu]Lu-PSMA-I&T. In accordance with the uptake data, the absorbed dose in the kidneys was significantly higher for [^177^Lu]Lu-PSMA-I&T compared with [^177^Lu]Lu-PSMA-617 (Fig. 4f).

### [^177^Lu]Lu-PSMA-I&T demonstrated significantly higher kidney binding during ex vivo and in vitro autoradiography studies compared with [^177^Lu]Lu-PSMA-617

**Fig. 5:**
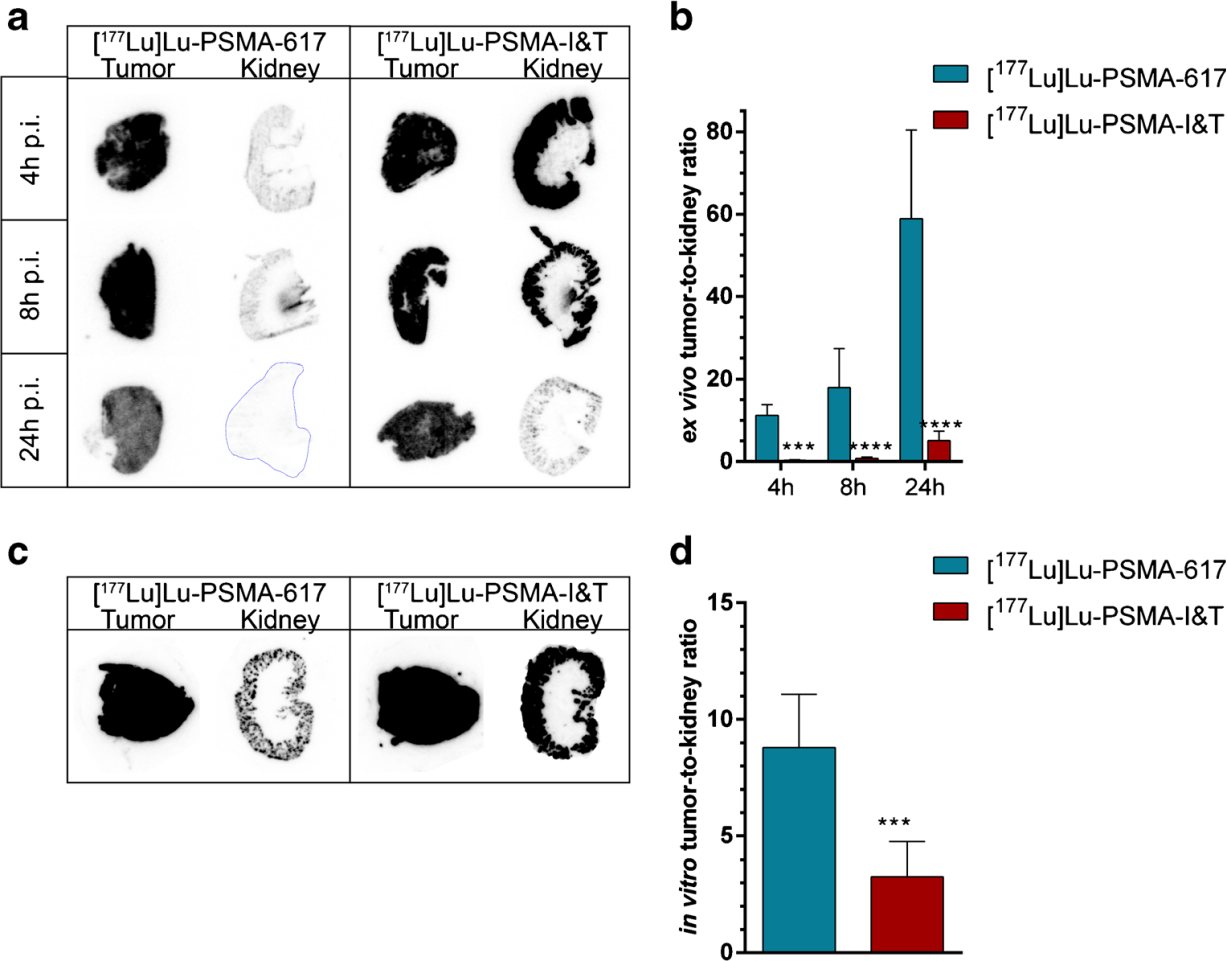
Ex vivo and in vitro autoradiography of PC295 PDXs and murine kidneys. **a** Representative images of ex vivo autoradiography results of mice injected with [^177^Lu]Lu-PSMA-617 or [^177^Lu]Lu-PSMA-I&T at 4, 8, or 24 h p.i. and **b** corresponding tumor-to-kidney ratio. **c** Representative images of in vitro autoradiography results of PDX and murine kidney tissue and **d** corresponding tumor-to-kidney ratio. (All error bars indicate standard deviation). Asterisks indicate significance (***P ≤ 0.001****P ≤ 0.0001)

Ex vivo autoradiography studies on tumor and kidney cryosections retrieved from mice of the biodistribution studies showed that radioactivity was homogeneously distributed throughout the PC295 PDX tissue for both tracers. Radioactivity in the kidney was only observed in the renal cortex (Fig. 5a). In accordance with the biodistribution data, tumor-to-kidney ratios were significantly lower for [^177^Lu]Lu-PSMA-I&T compared with those of [^177^Lu]Lu-PSMA-617 at all timepoints (Fig. 5b).

Subsequently, we compared the in vivo tracer uptake (measured ex vivo) with the results from in vitro autoradiography assays, using PC295 PDX and kidneys of untreated mice. In line with the ex vivo findings, a high level of homogenous tracer binding was found in the PC295 PDX as well as in the renal cortex (Fig. 5c). Again, [^177^Lu]Lu-PSMA-I&T revealed significantly higher level of binding in the kidney compared with [^177^Lu]Lu-PSMA-617, resulting in a significantly lower in vitro tumor-to-kidney ratio (Fig. 5d).

### Lutetium-177-labeled PSMA-617 and PSMA-I&T induced DNA double-strand breaks in vivo

**Fig. 6:**
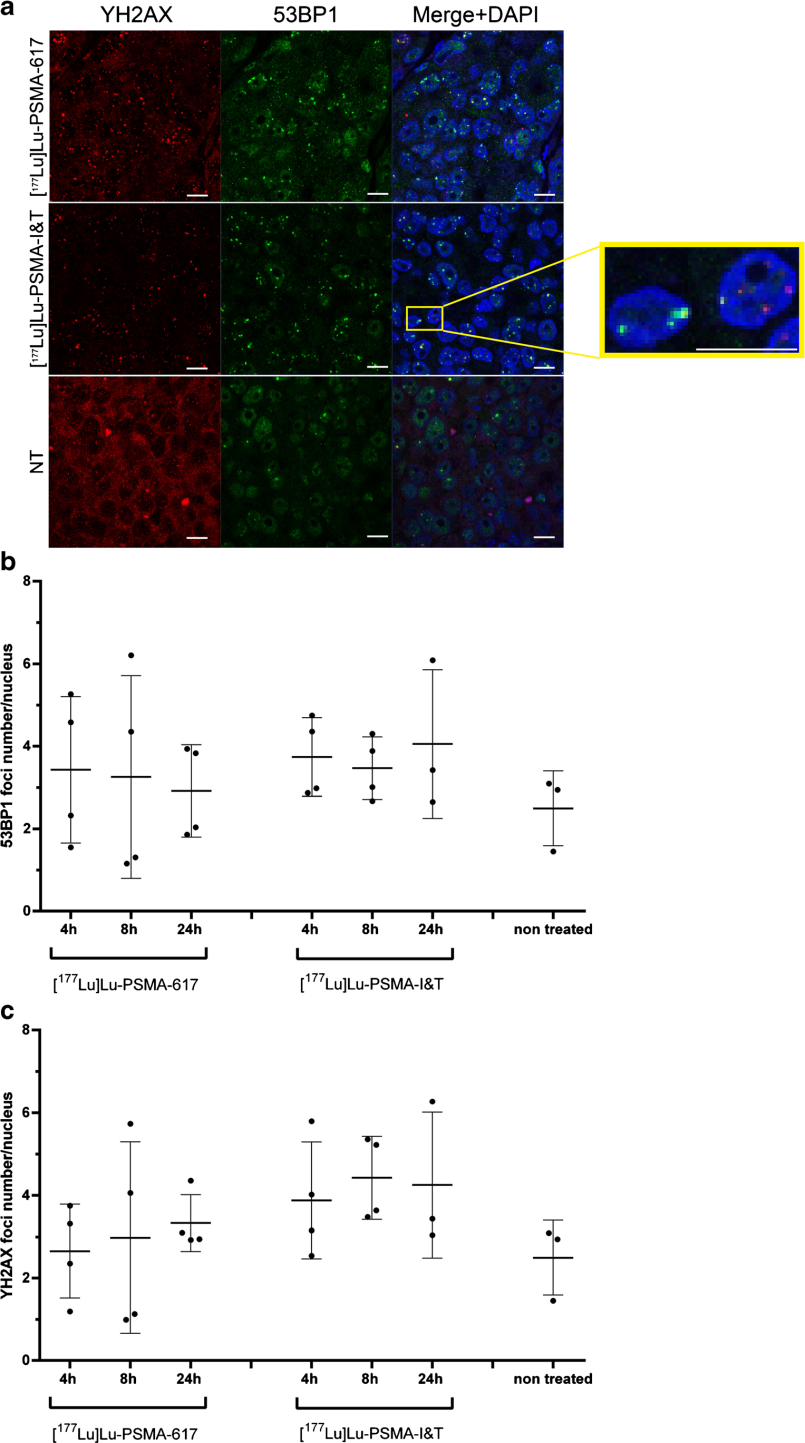
Ex vivo DNA double-strand breaks analysis on PC295 PDX tissue. **a** Representative images of tumor sections of mice injected with [^177^Lu]Lu-PSMA-617 or [^177^Lu]Lu-PSMA-I&T at 4 h p.i. and non-treated animals (scale bar = 10 μm). **b** Corresponding 53BP1γH2AX foci analysis. Average number of foci per nucleus per mouse is depicted. **c** Corresponding γH2AX foci analysis. Average number of foci per nucleus per mice is depicted. All error bars indicate standard deviation

[^177^Lu]Lu-PSMA-617 and [^177^Lu]Lu-PSMA-I&T both induced double-strand breaks (DSBs) in the PC295 PDX tumors as reflected by the trend of more 53BP1 and γH2AX nuclear foci at 4, 8, and 24 h p.i. compared with non-treated mice (Fig. 6). Even though a clear trend was observed of DSB induction, no significant difference in the number of DSBs between both tracers or the different timepoints was observed (Fig. 6 b, c). A large heterogeneity in average DNA damage induction levels between mice of one group and also within tumors was observed (Fig. 6b, c, Suppl. Fig. 2). 53BP1 and γH2AX foci did not always overlap; however, a clear correlation was observed between the level of both DSB markers in the tumors (Suppl. Fig. 2).

## Discussion

PSMA has become one of the most researched and promising PCa targets in the last decade, for the improvement of (metastasized) prostate cancer care. The focus of preclinical research has predominantly been on altering the chemical structure of the tracers, creation of bi-ligands (ligands that are specific for two targets), and the use of different therapeutic radionuclides to improve PSMA-TRT [9] resulting in an overwhelming number of novel radiolabeled PSMA-targeting tracers. Evaluation and comparison studies on existing high potential tracers are urgently needed to further support selection of PSMA-targeting tracers for theranostic purposes. Currently, PSMA-617 and PSMA-I&T are in clinical development to evaluate their use in PSMA-TRT [28, 29]. However, direct preclinical comparison of their therapeutic efficacy and safety is lacking. Increasing our knowledge regarding the binding characteristics of these tracers, both in tumor and healthy tissues, may aid future use and development of PSMA-TRT to make it safer and even better [10, 12].

We showed that the three tested PSMA-targeting tracers bind specifically to PSMA in vitro and possessed good binding affinity with IC_50_ values in the nanomolar range, in accordance with previously reported IC_50_ values [30, 31]. The inability of PSMA nanobody JVZ-007 to block or be blocked by the two small molecule inhibitors might indicate that JVZ-007 binds to a different epitope of the PSMA protein, which remains to be elucidated. The extremely low level of binding on the human renal and salivary gland tissues could be caused by this alternative domain binding of the nanobody and therefore could be of interest when developing novel tracers. However, the low tumor binding of PSMA nanobody JVZ-007 in vitro makes it in its present form not suitable as a therapeutic tracer. The significantly lower IC_50_ value of PSMA-617 suggests that PSMA-617 has a higher PSMA binding affinity compared with PSMA-I&T in vitro. Despite this, PSMA-617 and PSMA-I&T showed similar binding capacities to the PC295 PDX tumor both in vivo and ex vivo. The underlying reason for this discrepancy between models warrants further investigation. The similar uptake of [^177^Lu]Lu-PSMA-617 and [^177^Lu]Lu-PSMA-I&T was also reflected by a comparable DNA damage induction pattern in these tumors. The heterogeneity of DNA damage in one tumor and between animals of the same group in our study has also been observed in previous studies of other groups and might be explained by variations in PSMA expression levels in the tumors or differences in bioavailability [32].

In order to further assess the potential value of each tracer, their binding to healthy tissues was evaluated. Murine studies are not suitable to assess salivary gland binding because of the very limited (or absence of) uptake of PSMA-targeting tracers in salivary glands in different mouse models [33]. We showed that both in human renal and salivary gland tissue, as well as murine kidneys, [^177^Lu]Lu-PSMA-I&T displayed a significantly higher binding compared with PSMA-617*.* Ex vivo and in vitro autoradiography studies on PC295 PDX and murine kidneys suggest that the higher uptake of [^177^Lu]Lu-PSMA-I&T may not (only) be due to differences in pharmacokinetics between the two radiotracers, but also to a higher level of either specific or unspecific kidney binding.

Thus, although PSMA-617 and PSMA-I&T show similar binding characteristics on prostate tumors, our results indicate that PSMA-I&T has a less favorable tumor-to-kidney ratio than PSMA-617 as a higher binding to healthy organs may lead to an increased risk of toxicity. Previous preclinical studies also showed that [^177^Lu]Lu-PSMA-I&T and [^177^Lu]Lu-PSMA-617 have comparable tumor uptake 1 h after injection, with the latter having a slightly higher uptake [31, 34]. However, it must be noted that these were independent studies by different groups and even though the tumor model was the same, different mouse strains were used which could influence tumor uptake levels. Furthermore, to the best of our knowledge, there is no clinical trial in which PSMA-I&T and PSMA-617 are directly compared. Two separate clinical imaging trials of [^68^Ga]Ga-PSMA-617 and [^68^Ga]Ga-PSMA-I&T reported that the mean standardized uptake value (SUVmean) of the salivary glands of these patients was similar for both tracers [28, 35]. However, the SUVmean values of the kidneys of these patients 1 h p.i of PSMA-I&T were almost twice as high compared with that of PSMA-617. These data on renal uptake are in line with our preclinical in vivo biodistribution and in vitro autoradiography data, underlining the translational value of our study.

Literature showed peak renal uptake in mice of lutetium-177-labeled PSMA-617 value at 15 min p.i. and complete clearance at 4 h p.i. [29]. Hence, we have to take into account that we might have missed the tracer uptake in the murine kidneys in the first 4 h of the biodistribution, which has led to an underestimation of the calculated absorbed dose. This would explain the minimal kidney uptake of PSMA-617 in our in vivo biodistribution study compared with the high (early) renal uptake reported in patients [35]. Furthermore, future studies should include later timepoints (> 24 h p.i.) for a better estimation of the total dose. Despite this, the differences we found in the binding in vitro, in vivo, and ex vivo between PSMA-617 and PSMA-I&T on the salivary gland and renal tissues are substantial and should be considered when choosing a compound for patient PSMA-TRT.

The difference between both small molecule inhibitors with regard to composition and length of the linker is most likely the reason for the difference in binding. Various examples in literature highlight the impact of linker composition on binding characteristics and biodistribution of PSMA-targeting compounds [9]. For example, the replacement of the 2-naphthylalanine amino acid of the linker of PSMA-617 with the 3,3-diphenylalanine amino acid (creating HTK01167) led to a 6× lower renal binding and a 2× lower tumor binding [36]. Our data seem to support these observations indicating that the selection of linker composition seems essential in designing novel radiopharmaceuticals.

To conclude, we here show that [^177^Lu]Lu-PSMA-617 has more favorable binding and biodistribution characteristics in PSMA-positive cells, in healthy human salivary gland and renal tissues, and in mice bearing PSMA-expressing PDXs as compared with [^177^Lu]Lu-PSMA-I&T. This and future preclinical research is fundamental to select the most optimal PSMA-specific tracer to improve overall therapeutic efficacy of PSMA-TRT.

## Electronic supplementary material

ESM 1 (DOCX 11 kb)

ESM 2 (PNG 6377 kb)

High resolutin image (EPS 172230 kb)

ESM 3 (PNG 6377 kb)

High resolution image (EPS 171584 kb)

## Data Availability

Please contact the corresponding author.
