# Supplementary material for: Extensive preclinical evaluation of lutetium-177-labeled PSMA-specific tracers for prostate cancer radionuclide therapy
Source: Eur J Nucl Med Mol Imaging. 2020 Oct 23;48(5):1339–50. doi: 10.1007/s00259-020-05057-6 (PMC8113296; doi:10.1007/s00259-020-05057-6)
Supplement: Supplementary file 1 — (DOCX 11 kb) [file 259_2020_5057_MOESM1_ESM.docx]

**Supplemental figures**

**Suppl. Fig. 1.** IC**_50_** curves of PSMA-617, PSMA-I&T and JVZ-007 during in vitro displacement assays using DU145-PSMA #18, LNCaP and U2OS-PSMA cells The error bars represent the standard deviation.

**Suppl. Fig. 2.** *Ex vivo* DNA double strand breaks analysis on PC295 PDX tissue. Each dot represents the number of 53PB1 (a) or γH2AX (b) foci per nucleus. All error bars indicate standard deviation.
