# Supplementary figures and images for: Extensive preclinical evaluation of lutetium-177-labeled PSMA-specific tracers for prostate cancer radionuclide therapy
Source: Eur J Nucl Med Mol Imaging. 2020 Oct 23;48(5):1339–50. doi: 10.1007/s00259-020-05057-6 (PMC8113296; doi:10.1007/s00259-020-05057-6)

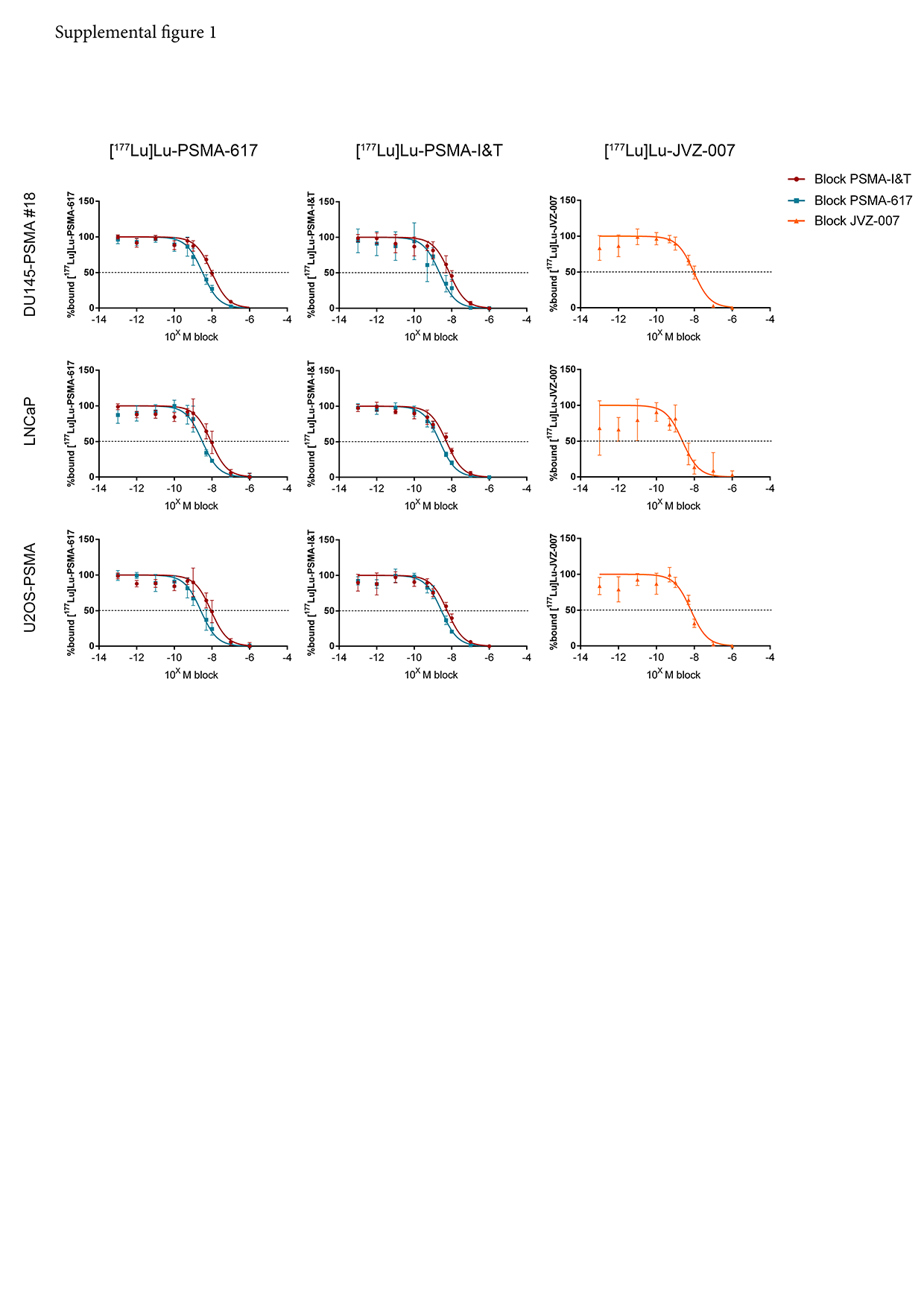

Supplement: Supplementary file 2 — (PNG 6377 kb) [file 259_2020_5057_Fig7_ESM.png]

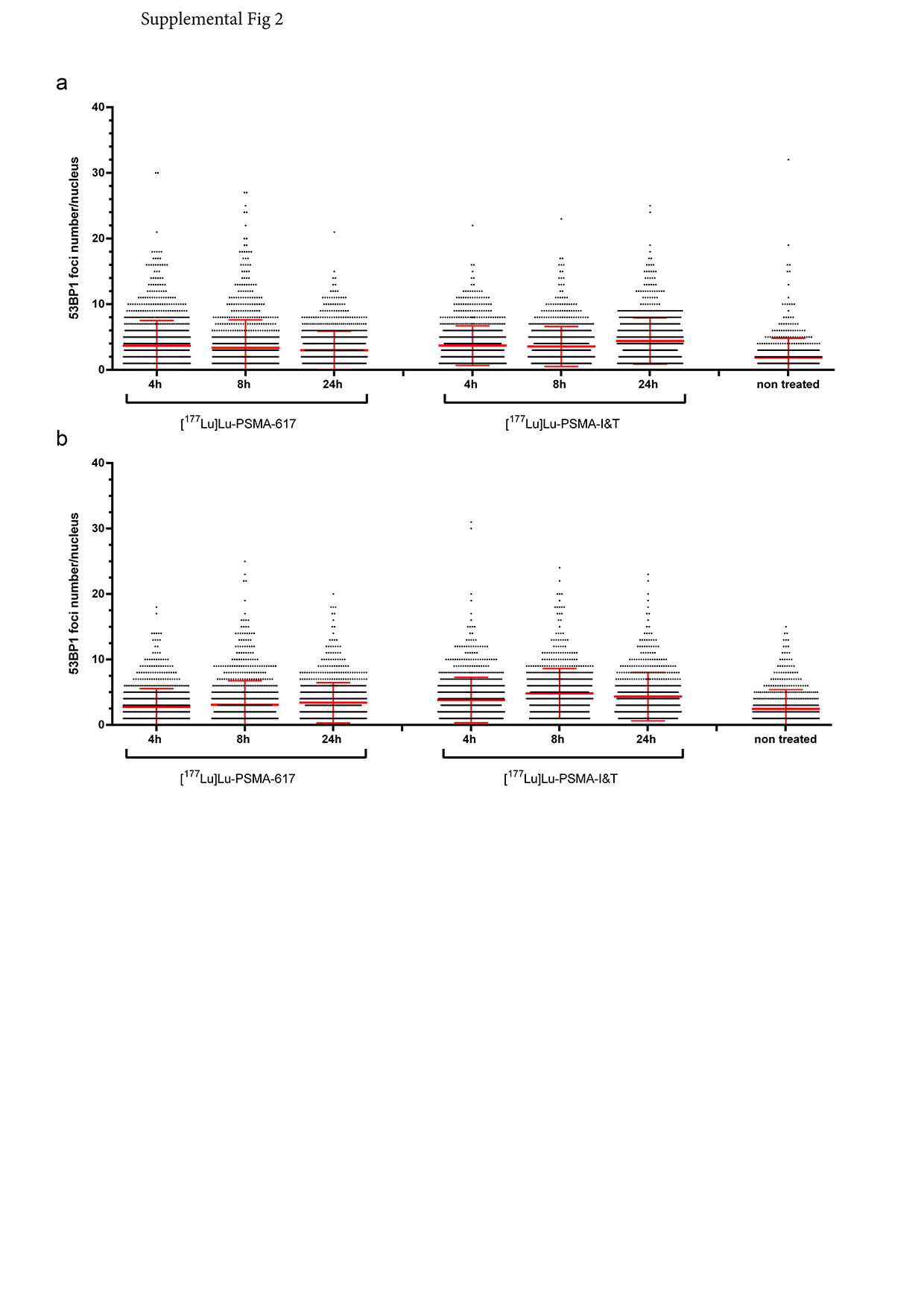

Supplement: Supplementary file 4 — (PNG 6377 kb) [file 259_2020_5057_Fig8_ESM.png]
